# Functional studies of GWAS variants are gaining momentum

**DOI:** 10.1038/s41467-020-20188-y

**Published:** 2020-12-08

**Authors:** Florence Lichou, Gosia Trynka

**Affiliations:** 1 Wellcome Sanger Institute, Wellcome Genome Campus, Cambridge, UK; 2 Open Targets, Wellcome Genome Campus, Cambridge, UK

**Keywords:** Functional genomics, Genome-wide association studies

## Abstract

Rapidly advancing genomic technologies and cross-disciplinary partnerships are accelerating the biological and clinical interpretation of genome-wide association studies, with some therapies developed based on these findings already being tested in clinical trials. The next decade promises further progress in understanding the function of genetic variants.

In the past 15 years, genome-wide association studies (GWAS) have been established as a default tool for mapping the genetic variation underlying complex traits. These studies have associated thousands of variants with hundreds of phenotypes, furthering our understanding of the genetic architecture of complex diseases^1^. Deciphering how associated variants modulate disease risk and severity, and how they impact cellular phenotypes informs mechanistic therapeutic hypotheses resulting in more effective drug discovery^2^. As the available genomic tools become more sophisticated and the cellular disease models more relevant, the next decade will deliver significant advancement in biological, clinical and therapeutic insights from GWAS variants. Accelerating these efforts will be achieved through close collaborative efforts and the blending of broad-scope systematic approaches with deep dives that rely on tailored assays in the context of specific biological processes. Here, we focus on some of the experimental approaches that will propel the translational impact of GWAS signals towards deeper pathomechanistic insights and improved therapies (Fig. 1).

## Interpreting variants at tissue and single-cell resolution

The vast majority of GWAS variants fall into non-coding regions and often overlap enhancers, promoters and open-chromatin regions, suggesting a role in regulating gene expression which can be highly cell-type and tissue-specific. Therefore, it is critical to perform functional studies in a relevant system. However, for many diseases, these pathologically relevant cell types and cell states are unknown. Computational approaches that integrate single-nucleotide polymorphisms (SNPs) with chromatin marks or with gene expression help to identify the tissues and cell states through which the risk variants contribute to the disease. Such SNP enrichment methods have successfully guided the identification of disease-relevant cell types and have highlighted the role of unexpected pathogenic cell types, such as microglia in Alzheimer’s disease^3^, or helped to refine disease-causal cell types and cell states, such as the role of early activation of CD4^+^ memory T cells in immune-mediated diseases^4^. In parallel, single-cell technologies, particularly for transcriptomics and chromatin accessibility (ATAC-seq), have revolutionised our understanding of cell biology providing critical insights into the heterogeneity of primary tissues in health and disease. Emerging single-cell data from all human tissues, alongside the development of statistical methods, will allow us to zoom deeper into the precise disease-causal cell types and processes.

Once the disease-relevant cellular context is defined, a combination of statistical and functional approaches is required to fine-map GWAS loci to identify causal variants, genes and mechanisms. Statistical approaches include colocalisation of disease variants with quantitative trait loci (QTL) for chromatin activity and gene expression to identify signals driven by the same causal variants^5^. Expanding existing QTL maps of chromatin and gene expression regulation with additional healthy tissue types and disease states, and across a range of in vitro-induced conditions, will continue to provide a critical resource for mapping genetic effects on molecular phenotypes. As costs of single-cell transcriptome profiling decrease, this technology will become more broadly amenable to expression QTL (eQTL) studies^6^, creating further opportunities to gain invaluable insights into genetic regulation of cellular heterogeneity and cell–cell interactions.

**Fig. 1:**
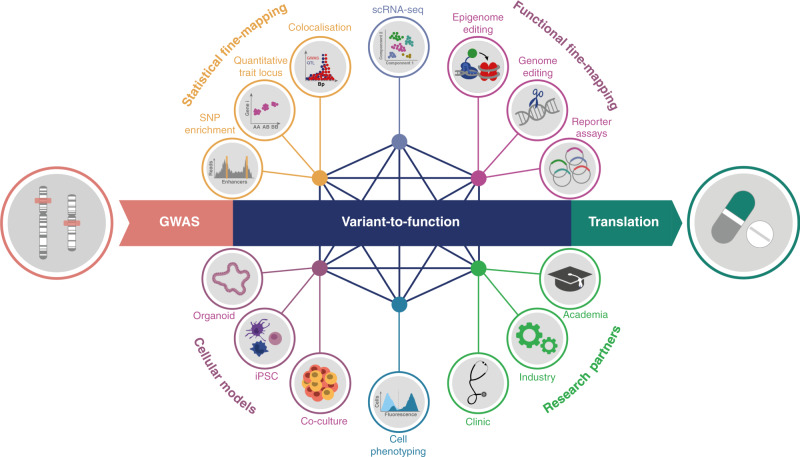
Variant-to-function approaches for the interpretation of genome-wide association studies (GWAS) signals. Meaningful interpretation of GWAS signals is rapidly advancing as available genomic tools become more sophisticated and the disease cellular models more relevant. Combining different approaches and closer cross-disciplinary collaborations strongly position the community to deliver significant advancement in biological, clinical and therapeutic insights from GWAS variants in the next decade.

## Developing accurate cellular in vitro models

The development of cellular models that closely reflect the physiological and disease context is critical for future translational approaches. The tremendous progress in differentiating human-induced pluripotent stem cells (hiPSC) into a range of cell types now enables the exploration of the effects of genetic variation and gene expression regulation in tissues previously inaccessible to in vitro experiments. Although in vitro cellular models do not perfectly capture the biology of primary tissues, their accuracy is rapidly increasing. This is further facilitated by the resolution of single-cell transcriptomics which has started to uncover previously unappreciated cellular heterogeneity in cell cultures; it allows for cell fate tracing and the monitoring of downstream effects of cell culture manipulations at the level of individual cells. This granularity also enables multiplexing, thus providing an enormous advantage for scaling up experiments and increasing reproducibility: cells from multiple individuals can be pooled, differentiated or stimulated together, and—following sequencing—be assigned to individual donors or culture conditions using genetic variants or barcodes. This opens up opportunities for population-scale in vitro experiments. Finally, it is now possible to use sophisticated cell culture models that recapitulate a diversity of different cell types and cell–cell interactions through organoid^7^ and human Organs-on-Chips^8^ systems that can mimic complex organs, such as the liver, gut or brain.

Technical challenges and high costs of such complex models currently restrict their utility to modest sample numbers. However, multiplexing capabilities and cell culture miniaturisation will help to overcome these limitations. Combined with high-throughput cell phenotyping assays and genomic technologies, sophisticated in vitro models will represent a critical tool to link genetic variants with intermediate cellular phenotypes.

## Dissecting GWAS loci with the genome-editing toolbox

Rapidly advancing genome-editing technologies are becoming a powerful complementary approach for dissecting the effects of genetic variants and providing important capabilities for both forward screens and validation. CRISPR screens (knock-out, activation (CRISPRa) and interference (CRISPRi)) coupled with high-throughput sequencing and massively parallel reporter assays (MPRAs) will catalogue the effects of regulatory elements and non-coding variants across different cell types, facilitating the identification and validation of causal variants and mechanisms. Bourges et al., for example, tested immune disease variants in 14 gene deserts that overlap super-enhancers in CD4^+^ T cells for their regulatory activity using MPRAs^9^. This revealed an immune disease-associated variant that disrupts NF-κB binding and, as a consequence, reduces the expression of *TNFAIP3* leading to increased T-cell-driven inflammation.

Complementary approaches can include tiling of enhancers at GWAS risk loci. Simeonov et al. used CRISPRa complexes to target a 178 kb region associated with immune diseases that regulate *IL2RA* expression^10^. This study highlighted six new regulatory regions associated with *IL2RA* upregulation and one of these regions was found to contain a variant associated with type 1 diabetes and Crohn’s disease. CRISPR screens combined with single-cell RNA sequencing (scRNA-seq) are a powerful tool for mapping thousands of enhancers to target genes while maintaining cell-type-specific context of gene expression regulation. A recent study used CRISPRi coupled with scRNA-seq to target over 5900 human candidate enhancers and identified 664 enhancer-gene pairs with evidence of regulatory interactions^11^.

Even though CRISPR technologies are improving our understanding of the role that genetic variants play in disease they currently suffer from important limitations. Some of the disease-relevant cell types may not be amenable to genome editing, the efficiency of edits may not be sufficiently high and the genome-editing perturbations may induce non-biological effects. However, with developments that make these technologies more precise, applicable to a broader range of cell types and scalable, the simultaneous testing of thousands of GWAS signals in a relevant cellular context is within reach.

## Constructing prospective catalogues of variant effects

To facilitate the interpretation of unknown variant effects, prospective variant catalogues can be created: possible variants are experimentally engineered and their effects on molecular and cellular phenotypes, such as cell viability, the activity of signalling pathways, cell response to external stimuli, protein abundance or drug toxicity are measured.

An example of how such catalogues could inform functional interpretation is illustrated by the *PPARG* locus, which is associated with risk for type 2 diabetes. While ~0.2% of the general population carry a *PPARG* missense variant, only 20% of these variants are associated with diabetes. Thus, to inform the future diagnostic interpretation of novel sequence variants, all 9595 possible PPARG amino acid substitutions were tested for their effects on the expression levels of the canonical PPARγ target, CD36, in a pooled phenotypic screen^12^.

Currently, such approaches are limited to a small number of genes and cell lines and are coupled with only a limited number of cellular phenotypes, e.g., use of haploid cell lines and cell death as a readout. However, technology advances will enable the precise introduction of variants at scale, in native cellular contexts and linked to complex cellular readouts, enabling a comprehensive survey of the effects of variants across large numbers of genes. The Alliance of Variant Effects (www.varianteffect.org) is bringing together researchers with diverse expertise to tackle these challenges in a concerted effort.

## Informing therapeutic intervention window from allelic series

Coding variants only account for a small proportion of GWAS signals, but successful functional studies increasingly provide valuable insights into disease biology highlighting their translational potential. One example is the rare coding variant rs34536443 in *TYK2*, which has a protective effect in a number of immune diseases (including multiple sclerosis, ankylosing spondylitis, ulcerative colitis and Crohn’s disease)^13^. The variant causes a proline-to-alanine substitution in the kinase domain of the TYK2 protein resulting in a weaker response of CD4^+^ T helper 1 and 17 type cells to proinflammatory signals. Despite the reduced T-cell activation response, individuals carrying rs34536443 protective alleles do not have an increased risk for infections or an otherwise impaired immune system, whereas individuals with complete *TYK2* loss of function develop immunodeficiency. Such an allelic series provides a natural genetic “dose-response curve”, suggesting that reducing the activity, but not complete inactivation of TYK2, may be an opportunity for intervention in immune diseases. Indeed, a selective TYK2 inhibitor is currently being tested in numerous clinical trials for the treatment of various immune diseases (https://www.targetvalidation.org/evidence/ENSG00000105397/EFO_0000540).

Although examples such as the *TYK2* coding variant are still rare, the increased power of upcoming large-scale case–control and biobank sequencing studies will enable the identification of more rare coding variants. Therefore, fast interpretation of coding variants will be critical in progressing swiftly from variant identification to function and potential therapeutic intervention.

## Joining forces across disciplines to accelerate progress

The progress in advancing disease cellular models combined with the increasingly precise genome-editing toolkit is rapidly enhancing the translational value of experimental in vitro studies. Our ability to more accurately interpret genetic signals and understand their mechanism of action will help with the development of tailored therapeutic approaches, driven by academic research and pharma industry alike.

The next 10 years will undoubtedly deliver a further significant increase in the number of phenotype-variant associations from GWAS. Fully harnessing the translational value of these association signals towards improved and safer therapies will require interdisciplinary efforts. Private–public partnerships such as Open Targets (www.opentargets.org), Accelerating Medicines Partnership (www.nih.gov/science/amp/index.htm) or the International Common Disease Alliance (www.icda.bio) bring together a diversity of expertise including genetics and genomics, clinical, molecular, and pharmacology, to jointly accelerate the progress of variant-to-function efforts.
